# Specialized marine exploitation on African islands: A multiproxy archaeological analysis of the Playa Chica site, Gran Canaria (11th–13th CE)

**DOI:** 10.1371/journal.pone.0349347

**Published:** 2026-06-10

**Authors:** Jonathan Santana, Jacob Morales, Simon-Pierre Gilson, Aitor Brito-Mayor, María del Carmen González-Ruiz, Paloma Vidal-Matutano, Miguel del Pino-Curbelo, Idaira Brito-Abrante, Pedro Henríquez-Valido, Javier Cruz Viera, Alberto Lacave-Hernández, Carmen Rodríguez Santana, Sandra Cancel, Marta Moreno-García, Amelia Rodríguez-Rodríguez

**Affiliations:** 1 G.I. Tarha. Arqueología y Patrimonio, Department of Historical Sciences, University of Las Palmas de Gran Canaria, Las Palmas de Gran Canaria, Spain; 2 SIIM Research Group, Department of Geography and History, University of La Laguna, La Laguna, Spain; 3 Department of Prehistory, University of Alcalá, Alcalá de Henares, Spain; 4 Casa Museo de Colón, Las Palmas de Gran Canaria, Spain; 5 Institute of History, CSIC, Madrid, Spain; Universidad de Sevilla, SPAIN

## Abstract

The Canary Islands, settled by Berber communities from Northwest Africa during the first millennium CE, offer a privileged window into coastal economies and early maritime adaptations that remain poorly documented on the adjacent mainland. Playa Chica (*Sardina*, *Gran Canaria*, Canary Islands) preserves a five-phase Indigenous occupation sequence spanning from the 6^th^ to the 13^th^ centuries CE. This study aims to test the practice of specialized marine exploitation during Phase 5 (11^th^–13^th^ centuries CE) employing a multiproxy approach. Systematic sampling and processing of sediments has provided a large assemblage dominated by molluscs and echinoids, followed by fish remains and crustaceans. Abundant fish scales indicate intensive on-site processing. The bone industry encompasses worked goat horn cores and hundreds of horn flakes interpreted as scaling tools, along with small hooks crafted from pig canines. Seed remains include crops (barley, durum wheat, fig) and smoke-prone plants used as fuels (*Euphorbia* sp., rhizomes of *Cyperus* sp., and elements of *Pinus canariensis* cones), consistent with fish drying or smoking. Low-intensity, smoke-prone fuels identified through anthracological analyses, and twenty-nine hearths reinforce this interpretation. Pottery is scarce and functionally associated with cooking, while lithics are abundant and largely locally sourced. Collectively, these findings define an activity area dedicated to the processing and preservation of marine resources, suggesting integration within coastal-inland exchange networks. As one of the most extensively sampled coastal contexts in the archipelago, Playa Chica provides critical comparative data for understanding the intensification of marine economies among Northwest African-derived populations during the late Holocene.

## 1. Introduction

Coastal exploitation and the processing of marine resources have long been central to the economic strategies of island societies [1,2]. This prominence reflects both the high productivity and relative predictability of intertidal and coastal ecosystems, and the structural constraints imposed by limited terrestrial biomass on many islands. Archaeological, isotopic, and ethnohistorical evidence consistently show that marine resources have been providing not only day-to-day subsistence, but also surplus, resilience, and social value in insular economies [3–5]. Despite Africa’s extensive coastline, adaptations to coastal environments during the late Holocene remain remarkably understudied [6,7]. The formative processes by which African human groups transitioned into maritime societies are still poorly understood. Recent research has identified offshore islands as critical locations for investigating early maritime adaptations, particularly in the Indian Ocean region [7,8]. Within this framework, the Canary Islands offer an exceptional comparative case study, providing a well-documented archaeological record of insular populations that developed sophisticated coastal adaptations and marine exploitation strategies. As the only Atlantic African archipelago colonized by Berber-speaking populations from the adjacent continent, the islands constitute a privileged window into understanding how Northwest African societies adapted to and exploited marine environments during the late Holocene.

**Fig 1:**
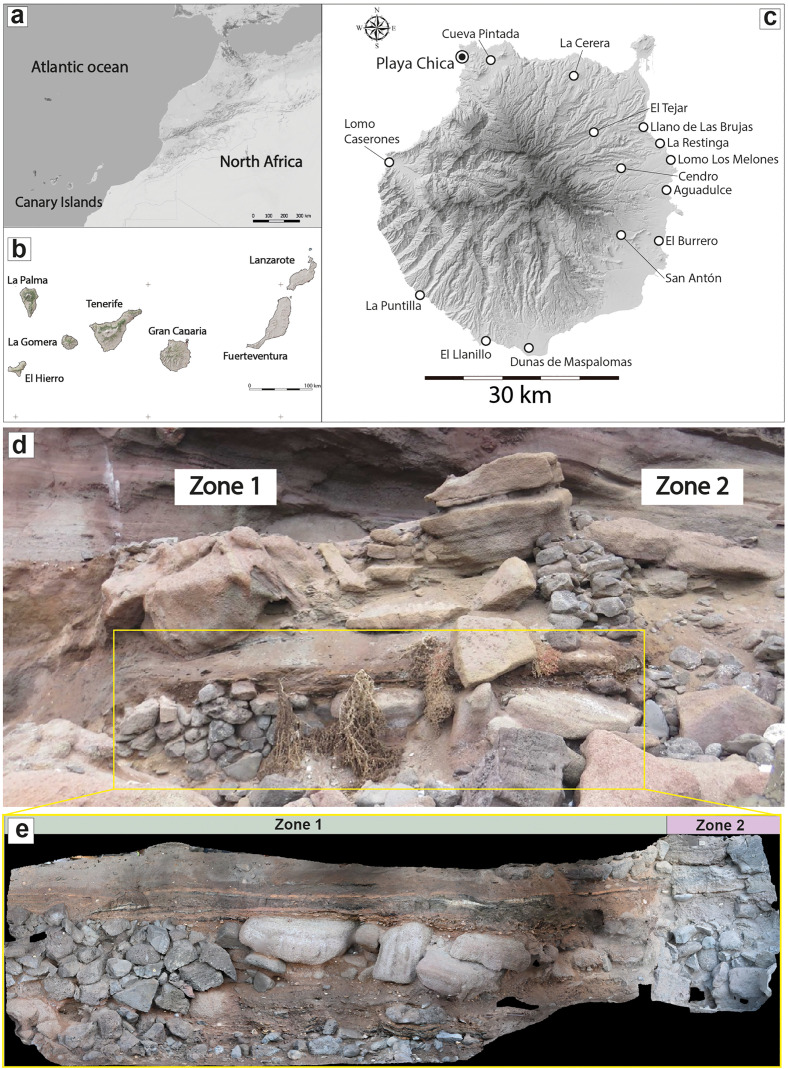
Location of Playa Chica on Gran Canaria, Canary Islands. **a)** position of the Canary Islands in Northwestern Africa; **b)** setting of Gran Canaria within the Canarian archipelago; **c)** place of Playa Chica in northwestern Gran Canaria and other sites cited within the text; **d)** general view of the site prior to archaeological intervention indicating Zone 1 and Zone 2. Large volcanic tuff blocks, detached from the cliff face, are visible across the area; **e)** western section of the site showing Zone 1 and the stone structure separating it from Zone 2.

This study presents the findings of archaeological research conducted at the pre-European site of Playa Chica (Sardina, Gran Canaria, Canary Islands) (Fig 1). The site’s archaeological deposits, approximately two meters in depth, provide significant evidence for early human settlement on the island’s coastline. Playa Chica reveals five distinct phases of occupation spanning from the 6^th^ to the 13^th^ centuries CE. Although archaeological features are well preserved, marine erosion has substantially impacted the stratigraphic sequence, resulting in the partial destruction of cultural deposits. This paper specifically addresses the site’s most recent Indigenous occupation (11^th^–13^th^ centuries CE), during which evidence of specialized marine-resource exploitation is attested.

The Canary Islands lie approximately 90 km off the northwest coast of Africa (Fig 1). From the 1^st^–3^rd^ centuries CE until the arrival of Europeans in the Late Middle Ages, the archipelago was inhabited by the Canarian Indigenous people [9]. Genetic, linguistic, and archaeological evidence indicates that this population derived from autochthonous Berber speaking groups in north-western Africa [10–13]. Throughout this paper, we use Indigenous to refer to the pre-European inhabitants of the Canary Islands, following established literature [13], while acknowledging ongoing discussions regarding terminology.

The islands offer abundant marine resources including shellfish, fish, seabirds, and seaweeds, as well as a diverse and productive terrestrial flora. In contrast, native land mammals and other medium- to large-sized land prey were scarce. By the time Berber speaking settlers arrived, the largest endemic vertebrates were lizards of the genus *Gallotia* (ca. 30–60 cm total length) and the now-extinct giant rats of the genus *Canariomys* (ca. 39–50 cm total length) [14,15]. Bringing their own traditions, the colonists introduced domestic crops and livestock, effectively implanting their lifestyle in the Islands [14,16–19]. Their economic strategies depended heavily on lithic technologies including rotary and saddle-quern hand-mills for cereal processing [20], and on a varied ceramic repertoire in which specialized cooking vessels played a central role [21].

Gran Canaria is the third-largest island within the Canarian archipelago. Its biogeography and climatic conditions have influenced settlement patterns and economic practices since initial colonization [22]. Located on the rugged northwestern coast, Playa Chica exemplifies how early Berber speaker settlements occupied the island’s shores to exploit coastal resources, including fish and shellfish. This pattern is also observed in other islands of the Canarian’s archipelago from the onset of their colonization [9]. This area is also characterized by cooler temperatures, higher precipitation, and more abundant vegetation compared to the island’s southeastern regions, which have sandy shores and arid climates [23,24].

The interaction between environmental constraints and cultural choices has significantly influenced local economic systems during the Indigenous period in Gran Canaria. The limited distribution of certain lithic materials, such as knappable volcanic glass fragments and outcrops of vesicular volcanic rocks used for milling, exemplifies natural limitations. These lithic resources were intensively exploited at select sites functioning as production centres or workshops embedded within settlement networks, facilitating the broad circulation of finished goods [25]. This pattern contrasts with more widely practiced crafts, such as pottery production and the manufacture of coarse-grained volcanic lithic tools, which occurred locally [26,27]. Settlement networks also included specialized, relatively remote activity areas where household-scale production of common goods, e.g., ceramics, likely took place.

Archaeological and historical evidence indicates that Gran Canaria’s Indigenous communities relied upon diverse subsistence strategies [16,22,28–30]. While animal husbandry (primarily goats and pigs) and cereal cultivation were widespread, marine resources such as fish and shellfish constituted significant dietary components, particularly in coastal settlements [28,31,32]. Archaeological evidence of marine-resource consumption predominantly derived from bioanthropological studies, including stable isotope analysis [32–35] and palaeopathological investigations [12,36–38]. Although specialized marine exploitation tools have been identified [31], comprehensive evidence of seafood processing and consumption contexts remains limited.

The identification of specialized marine-resource exploitation in archaeological contexts can be achieved by multiple, complementary methodological approaches. Previous studies in different geographical contexts have usually incorporated three primary categories of evidence [1]: (1) zooarchaeological analysis of faunal remains, such as fish bones, mollusc shells, and other marine invertebrates; (2) typological and functional analysis of tools and artifacts associated with fishing and shellfish gathering, including those made from bone, stone, or other raw materials; and (3) the identification of combustion features related to the processing and/or preservation of marine products (e.g., facilities used for smoking or drying). These categories specifically address animal marine exploitation and do not encompass other forms of marine resource use, such as the harvesting of seaweeds, seagrasses, or the extraction of sea by-products (e.g., salt). This study aims to research those three primary categories of evidence to examine the practices of specialized marine activities at Playa Chica archaeological site during the Berber period.

Despite the importance of marine resources and Gran Canaria’s 252 km coastline, few coastal archaeological sites have been systematically excavated using rigorous sampling methodologies [39]. Recent multiproxy research on shell middens in Tenerife, the biggest island of the Canarian archipelago, has revealed that coastal archaeological deposits can serve as archives of both Indigenous lifeways and long-term littoral environmental change. This research confirms the untapped potential of these contexts across the archipelago [40]. At Playa Chica, systematic sediment sampling and flotation were conducted to recover plant macro-remains and small archaeological materials, including fish remains. Playa Chica is a rock-shelter site perched above a sandy-cobble beach on the north-western coast of Gran Canaria. Although fishers reused the shelter during the 20^th^ century (and probably earlier), a sterile layer of disaggregated volcanic tuff protected the underlying Indigenous cultural deposits from disturbance. Consequently, Playa Chica represents one of the most thoroughly sampled archaeological contexts associated with marine activities in the Canary Islands, providing high-resolution data for examining continuities and changes in human–marine relationships.

## 2. Materials and methods

### 2.1. Materials

Three excavation seasons (2020–2022) were conducted at Playa Chica as part of a broader investigation into Berber colonization of the Canary Islands. The site was selected because rescue excavations had yielded radiocarbon dates pointing to early human occupation and preliminary observations revealed abundant marine faunal remains and artefacts associated with fishing activities in well-preserved stratigraphic contexts. These findings suggested that Playa Chica could provide high-resolution evidence of specialized coastal exploitation rarely documented in the archipelago. Fieldwork pursued three aims: (1) to obtain a stratigraphically controlled sample of archaeological remains by flotation of all sediments and collection of items through 5, 2, 1, and 0.5 mm meshes; (2) to refine the site chronology via high-precision AMS dating of identified, short-lived terrestrial samples; and (3) to recover cultural materials that would elucidate the range of activities performed at the shelter. Importantly, the preserved cultural deposits contained abundant archaeological remains in their primary depositional context, providing a direct record of past practices carried out at the site.

### 2.2. Zooarchaeological analysis

Marine and terrestrial faunal remains were recovered both in situ and after flotation of all excavated sediments using 1 mm mesh sieves. Specimens were quantified using standard zooarchaeological measures: total number of specimens (NSP), number of identified specimens (NISP), and minimum number of individuals (MNI). Taxonomic identifications were made by comparison with the materials hold at the Zooarchaeology reference collection (LPZ) in the University of Las Palmas de Gran Canaria, which includes specimens of mammal, fish, mollusk, and herepetofauna, and the ichthyoarchaeological collection stored at the Cueva Pintada Museum. Anatomical identifications followed Hillson [41] and Schmid [42] for mammals. Taphonomic observations, including butchery marks, thermal alteration, and post-depositional modifications, were recorded for all identifiable specimens.

### 2.3. Seeds analysis

All excavated sediments were processed using hand-bucket flotation with light fractions collected on 0.5 mm mesh. In total, 115 sediment samples, representing a combined volume of 457 litres, were analysed for plant macro-remains, including seeds and wood charcoals (anthracological analysis, see Section 2.4). Carbonized seeds and fruits were sorted under a stereomicroscope and identified using the modern seed reference collection housed at the University of Las Palmas de Gran Canaria and standard identification keys.

### 2.4. Anthracological analysis

Each wood charcoal fragment was fractured manually to expose transverse, tangential, and radial sections for taxonomic identification. Observations were made using a Brunel SP-400 bright/dark field incident light microscope at 50–500 × magnification at the University of La Laguna. Botanical identifications followed Schweingruber [43,44] and the modern charred reference collection from the Department of Geography and History, University of La Laguna. Photography and detailed anatomical observations were carried out using a Zeiss EVO MA 15 scanning electron microscope (SEM) at the General Research Support Service (SEGAI), University of La Laguna. Taphonomic features, including fungal degradation and vitrification, were also documented.

### 2.5. Ceramic and lithic analysis

Ceramic fragments were classified following the typological framework established by del Pino et al. [45] for pre-European Gran Canaria, with recording of fabric, firing atmosphere, surface treatment, and decoration. Lithic artifacts were analysed technologically, distinguishing between chipped (flakes, retouched pieces, cores, debris) and non-chipped (hammers, anvils, abrasive tools) categories. Raw material provenance was assessed based on macroscopic characteristics and comparison with known geological sources on the island. The descriptive protocol of use-wear traces has been detailed elsewhere [46]. A Nikon SMZ1000 stereomicroscope (8×–80×) and a Nikon Labophot-2 metallographic microscope operating at high magnification (100×–400×) with incident brightfield light were used for detailed observation of traces [47].

### 2.6. Spatial analysis

All archaeological features and artefacts were recorded in three dimensions using a Topcon IS-3 total station and integrated with structure-from-motion photogrammetry (Nikon D750; Agisoft PhotoScan). These datasets were combined in a GIS to support intra-phase spatial analyses of Phase 5, including density mapping and the assessment of activity-area structure. To evaluate spatial patterning in terrestrial faunal remains, we applied a density-based clustering approach (DBSCAN) implemented in QGIS 3.36.2, following standard formulations of the algorithm [48]. Analyses were conducted on two datasets: (i) faunal specimens plotted in situ during excavation (n = 163), and (ii) additional faunal remains recovered through sediment processing (n = 382). Because the latter lacks individual finds coordinates, those specimens were spatially represented by generating random points within the excavated polygons corresponding to their provenience units (random point-in-polygon procedure), enabling comparative, sensitivity-aware assessment of clustering patterns between the georeferenced and bulk-recovered assemblages.

### 2.7. Ethical statement

This study received approval from the Cultural Heritage Office of the Government of the Canary Islands, the authority responsible for overseeing the heritage of the Indigenous communities of the archipelago, including approval for publication of the results. Our research focuses exclusively on archaeological materials dating from the Indigenous period of the Canary Islands. Currently, there are no communities within the Canary Islands that identify themselves as direct descendants of the archipelago’s indigenous population. The preliminary results of the analyses have been disseminated to the local community in advance to their publication.

## 3. Results

### 3.1. Stratigraphic context

**Fig 2:**
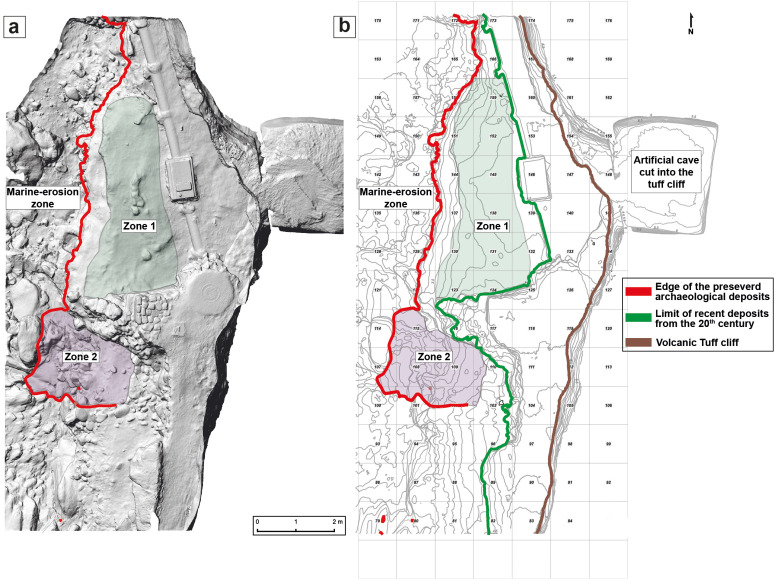
Playa Chica site. **a)** Aerial (plan) view of the excavation area, highlighting Zones 1 and 2. The sector significantly eroded by marine action is also marked, with the western section depicted in Fig 1e, where wave activity has truncated the stratigraphy and revealed the western profile; **b)** The same view is presented with the excavation grid system. The boundaries of the excavated area within each zone are indicated, along with the marine-cut scarp and the cliff line against which the archaeological deposits are preserved.

Playa Chica lies barely 3 metres from the present shoreline and preserves nearly 2 m of stratified deposits, although wave action has eroded a large portion of the original occupational surface. Marine erosion represents a serious and growing threat to this site and to other coastal archaeological sites across the Canary Islands [49]. The remaining sequence documents five occupation phases, interspersed with episodes of cliff collapse involving rock and volcanic-tuff blocks (Figs 1d and 2a). Anthropogenic layers contain architectural remains, activity floors, and well-preserved combustion features; they alternate with levels of dark beach sand (fine gravels derived from shelter-wall weathering) and organic accumulations generated by human activities.

Excavation was conducted in two areas, Zone 1 and Zone 2 (Fig 2a–2c), separated by a stone wall that subdivides the shelter near the upper part of the sequence:

*Zone 1.* Fieldwork here targeted the most recent occupation (Phase 5), sealed by the collapse of large tuff blocks from the cliff face. Radiocarbon data place this phase in the 11^th^–13^th^ centuries CE (Table 1). Activities were carried out on an uncompacted sand surface containing numerous hearths, including several large features reused multiple times.

**Table 1:** Radiocarbon dates from Playa Chica.

Zone	Phase	SU	Material	Species	LabID	CRA	Error	Calibrated (68%)		Calibrated (95%)		Method	Reference
1	5	2	Charred seed	*Pinus canariensis*	Beta-593528	870	30	1160	1220	1045	1265	AMS	Santana *et al*. 2024
1	5	2	Charred seed	*Laurus novocanariensis*	Beta-593527	880	30	1155	1220	1045	1230	AMS	Morales *et al*. 2023
1	5	5	Charred seed	*Triticum* cf. *durum*	GrM-35234	892	21	1055	1210	1050	1220	AMS	This study
2	3	1009	Charred seed	*Hordeum vulgare*	Beta-593529	1240	30	690	870	675	880	AMS	Morales *et al*. 2023
1	2	A27	Bone	*Sus s. domesticus*	Beta-457377	1370	30	640	675	600	775	AMS	Santana *et al*. 2024
2	1	A24	Bone	*Capra hircus*	Beta-457376	1460	30	590	645	560	650	AMS	Santana *et al*. 2024
2	1	1021	Charred seed	*Visnea mocanera*	GrM-35233	1475	21	575	635	565	645	AMS	This study

*Zone 2.* A 2 m² test pit exposed a markedly different stratigraphy, reaching the earliest occupations. The lower sequence primarily attests to predominantly domestic activities, as evidenced by ceramics, bone and stone tools, and refuse from the consumption of goats (*Capra hircus*) and pigs (*Sus scrofa domesticus*). Charred seeds of barley (*Hordeum vulgare*), wheat (*Triticum* cf. *durum*), lentil (*Lens culinaris*), broad bean (*Vicia faba*), and fig (*Ficus carica*) were also recovered [16]. Phase 5 is absent in this area.

Phase 4 exhibits a dry-stone structure with a prepared earthen floor that extends from Zone 2 into Zone 1 under the stone structure demarcating both zones. The floor appears to be a mixture of ground volcanic tuff, sand, and lime; forthcoming geoarchaeological analyses will refine this interpretation. In Zone 2, the structure’s interior yielded evidence of fire use, consumption of marine and terrestrial fauna, and lithic knapping. Phase 4 was not excavated in Zone 1. Although undated directly, stratigraphic relationships place it in the 9^th^–11^th^ centuries CE.

Phase 3, identified in profile in Zone 1 but not yet excavated, consists of compacted earth floors rich in artifacts and dates to the 7^th^–8^th^ centuries CE (Table 1). In Zone 2, it appears as a secondary fill of stones and artifacts that sealed a preceding collapse episode that had infilled the structure associated with Phase 2.

Phase 2 is marked in the northern wall of the Zone 2 test pit by a stone alignment perpendicular to the shelter wall, delimiting compacted floors and likely representing an architectural component. Radiocarbon assays assign Phase 2 to the 7^th^–8^th^ centuries CE (Table 1). A stone wall is visible in the section profile of Zone 1, which likely belongs to an architectural feature (Fig 2).

Phase 1 represents the initial human occupation, directly overlying the beach surface. It contains several fire episodes, remains of marine and terrestrial fauna, wild and domestic plants, and evidence of lithic knapping. AMS dating places this phase in the 6^th^–7^th^ centuries CE (Table 1).

### 3.2. The occupation of Phase 5

Phase 5 represents the most comprehensively documented occupation at Playa Chica, confined to Zone 1 and documented via open-area excavation during three archaeological seasons. Consequently, the remainder of this paper centres on that phase (Fig 2).

**Fig 3:**
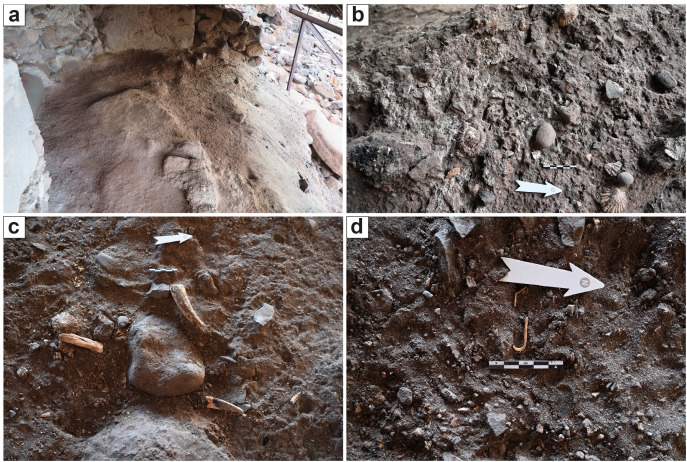
Playa Chica, Zone 1. **(a)** sterile upper layer of disaggregated volcanic tuff and black beach sand sealing Phase 5; **(b)** surface of SU 2, with a matrix dominated by black sand and featuring flat hearths, shellfish remains, lithic artefacts, and fish scales; **(c)** goat horns used as fish descalers in SU 2. The white particles are predominantly fish scales mixed within the volcanic black sand; **(d)** pig-tusk fishhook recovered in primary context within SU 2.

During the archaeological work, two primary archaeosedimentary deposits, Stratigraphic Units (SUs) 2 and 3, were identified within Zone 1, both sharing a comparable loose, sandy matrix. Both levels feature numerous hearths and a substantial quantity of marine faunal remains, including fish bone elements and scales, shellfish, echinoderms, and crustaceans (Fig 3). Additionally, they contain charred plant macroremains and a diverse artifact assemblage comprising lithic tools (Fig 3b, 3c), bone and tooth implements (Fig 3c, 3d), and associated manufacturing debris. In stark contrast to other coastal Indigenous sites on Gran Canaria [26,30,50–53], ceramics and terrestrial fauna bones are remarkably scarce. The unconsolidated matrix precluded the identification of discrete microfacies and clearly preserved primary depositional surfaces except in the case of combustion features. Although all finds were georeferenced, subsequent aeolian infilling and trampling appear to have blurred the boundaries of individual depositional episodes. In this respect, SU 2 and SU 3 are best interpreted as palimpsestic occupation deposits, formed through repeated episodes of use that accumulated within a stratigraphic context of limited internal resolution to detect depositional episodes.

Twenty-nine individual fireplaces were recorded within SU 2 and SU 3. Stratigraphic and spatial analyses indicate that many represent successive re-fires of 15 primary flat and shallow pit hearths. These hearths range from approximately 20–100 cm in diameter, defined by lenses of charcoal and ash resting directly on the sandy substrate or on previous hearths. Combustion structures (CS 1 in SU 2 and CS 10 in SU 3) dominate the assemblage, while smaller fire spots toward the north and south testify to diminishing heat intensity away from the activity core. Horizontal discontinuities formed by blockfalls and episodic water flow fragmented the surface of SU 2—and, to a lesser extent, SU 3—rendering the integrity of certain hearths compromised.

These deposits were subsequently overlain by SU 1, a culturally sterile deposit that corresponds to the uppermost sedimentary level documented during excavation. SU 1 is composed of aeolian beach sands and gravels originating from the disintegration of the volcanic tuff that constitutes the natural substrate of the rockshelter, along with blocks of tuff detached from the shelter roof. The unit exhibits a light brown to violet-brown colour and exhibits a thickening trend towards the eastern sector, while thinning towards the western and southern ends, where the archaeological sedimentary package is exposed in section. Notably, this level no longer contained modern refuse associated with recent utilization of the shelter (SU 0).

Immediately below, SU 2 constitutes the upper major archaeological sedimentary package within Zone 1. It comprises a loose volcanic sandy matrix characterized by a high density of archaeological material and abundant interstratified hearths. The sediment exhibits a generally dark brown to black coloration due to the black colour of the volcanic sand, as well as the presence of wood charcoal and ash resulting from repeated hearth utilization. Notably, reddish rubefaction is observed around the peripheries of combustion features. This unit is best interpreted as a palimpsest, as the low compaction and relative homogeneity of the sandy matrix impede the identification of distinct primary depositional surfaces associated with each fire event. Consequently, the internal stratigraphic sequence of SU 2 primarily relies on the relationships between hearths rather than on clearly separable depositional facies. Furthermore, SU 2 demonstrates significant lateral instability, particularly in the western sector, where downslope sediment movement generated erosive channels that penetrated the underlying package.

Beneath this upper palimpsest, the SU 3 forms the second major archaeological sedimentary package in Zone 1. This unit exhibits a more compact sandy matrix compared to SU 2 and displays greyish-red to reddish hues resulting from a combination of disintegrated tuff and sediment rubefaction. Similar to SU 2, its internal structure is strongly characterized by repeated combustion events, but the deposit is somewhat more sedimentologically compacted. SU 3 yielded a notably high density of lithic artifacts and fish scales, suggesting intensive fish processing and consumption. Additionally, a posthole in square 131C indicates the former presence of a light structure constructed from perishable materials. Several combustion features documented on or near its surface demonstrate that this unit records repeated occupation episodes comparable to those observed in SU 2, albeit under somewhat different sedimentary conditions.

Underlying SU 3, SU 4 is a light brown-orange, weakly compacted. It was not excavated extensively, but rather delimited during the excavation of overlying deposits. It contains very little archaeological material, and the few finds recorded are very small and may have percolated downward from SU 3. Its exposure in the northern sector also indicates that, in this area, later erosive events removed part of the overlying archaeological sequence before the formation of the upper natural fill.

The northern margin of Zone 1 is heavily truncated by marine erosion and subsequent block falls, reducing the preserved thickness of SU 2 and SU 3 and limiting our ability to reconstruct the full spatial extent and depositional dynamics of Phase 5 (Fig 2).

### 3.3. Radiocarbon dating of phase 5

Three accelerator mass spectrometry (AMS) radiocarbon determinations on charred seeds were obtained for Phase 5, analysed at Beta Analytic, Inc., Miami, and at the Centre for Isotope Research, University of Groningen. Two samples from SU 2 calibrate to 1120–1250 cal CE and 1050–1260 cal CE, respectively (Table 1). A third radiocarbon date from SU 3 yielded a date of 1050–1220 cal CE. All three measurements pass the Ward and Wilson [54] χ² test for contemporaneity (T’ = 0.4; T(0.05, df = 2) = 6.0), indicating that the associated human activities likely occurred within a single human generation.

### 3.4. Marine fauna

**Fig 4:**
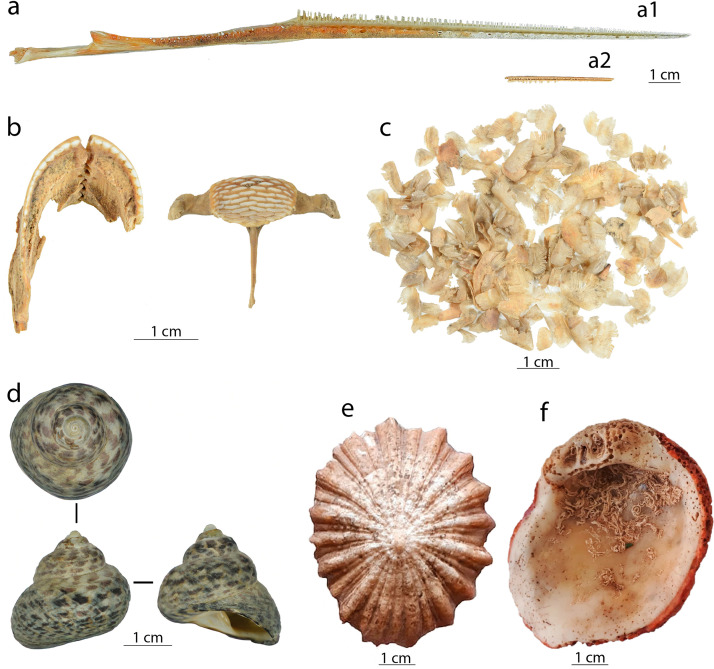
Marine faunal remains from Phase 5 in Zone 1, Playa Chica. **(a)** *Belone belone*—a1 shows a dental element from the reference collection, and a2 shows a fragmented dental element recovered at Playa Chica; **(b)** *Sparisoma cretense*; **(c)** fish scales; **(d)** *Phorcus sauciatus*; **(e)** *Patella ordinaria*; and **(f)** *Spondylus senegalensis*.

The assemblage comprises 19,615 specimens, of which 14,508 are molluscs, 3,672 echinoderms (sea urchins), 1,349 bony fish, 2 cartilaginous fish, and 84 crustaceans (Table 2). Eighteen mollusc taxa were identified: sixteen marine gastropods, one bivalve, and one scaphopod. Gastropods dominate the sample, with *Phorcus sauciatus* (Fig 4d) representing 33% of the total MNI. At the family level, Patellidae constitutes 47% of the MNI, followed by Trochidae (34%) and Muricidae (11%); all remaining families together account for 7% (Table 2). Bivalves are virtually absent, represented only by fragmentary valves of *Spondylus senegalensis* (Fig 4f). Scaphopods are similarly rare, with five broken individuals of *Antalis vulgaris* recorded. Among decapods, only the rock crab *Grapsus grapsus* is present, represented by 60 fragments. Sea urchin remains are abundant: *Diadema africanum* accounts for 2,707 elements (353 tests, 2,354 spines), while a further 965 fragments are identified only to Echinoidea.

**Table 2:** Marine fauna remains from Phase 5 of Zone 1, Playa Chica. Note: % NISP and % MNI calculated within each taxonomic class. (-) = not calculated for this group. Brachyura sp. MNI not calculated.

Family	Taxon	NISP	% NISP	MNI	% MNI
**Teleostei**					
**Belonidae**					
	*Belone belone*	501	69,8	29	34,5
**Carangidae**					
	*Trachinotus ovatus*	6	0,8	2	2,4
	*Trachurus trachurus*	6	0,8	2	2,4
**Clupeidae**					
	*Clupeidae* sp.	45	6,3	4	4,8
**Epinephelinae**					
	*Epinephelinae* sp.	6	0,8	2	2,4
	*Epinephelus marginatus*	3	0,4	2	2,4
	*Mycteroperca fusca*	4	0,6	2	2,4
**Haemulidae**					
	*Parapristipoma octolineatum*	3	0,4	1	1,2
	*Pomadasys incisus*	7	1	2	2,4
**Labridae**					
	*Thalassoma pavo*	2	0,3	1	1,2
**Muraenidae**					
	*Muraena sp.*	14	1,9	4	4,8
	*Muraena augusti*	2	0,3	2	2,4
	*Muraena helena*	1	0,1	1	1,2
**Pomacentridae**					
	*Similiparma lurida*	2	0,3	1	1,2
**Scaridae**					
	*Sparisoma cretense*	74	10,3	10	11,9
**Sebastidae**					
	*Helicolenus dactylopterus*	1	0,1	1	1,2
**Sparidae**					
	*Boops boops*	12	1,7	3	3,6
	*Diplodus sargus*	1	0,1	1	1,2
	*Diplodus cervinus*	2	0,3	1	1,2
	*Lithognathus mormyrus*	1	0,1	1	1,2
	*Oblada melanura*	1	0,1	1	1,2
	*Pagellus erythrinus*	1	0,1	1	1,2
	*Pagrus auriga*	2	0,3	2	2,4
	*Pagrus pagrus*	3	0,4	3	3,6
	*Sarpa salpa*	14	1,9	3	3,6
	*Spondyliosoma cantharus*	2	0,3	1	1,2
**Sphyraenidae**					
	*Sphyraena viridensis*	2	0,3	1	1,2
**Total Teleostei**		**718**	**100**	**84**	**100**
**Elasmobranchii**					
**Carcharhinidae**					
	*Carcharhinus plumbeus*	2	100	1	100
**Total Elasmobranchii**		**2**	**100**	**1**	**100**
**Echinoderms**					
**Echinoidea**					
	*Echinoidea sp.*	965	–	–	–
	*Diadema africanum*	2707	–	–	–
**Total Echinoderms**		**3672**	**–**	**–**	**–**
**Crustacea**					
**Brachyura**					
	*Brachyura* sp.	24	–	–	–
	*Grapsus grapsus*	60	–	8	–
**Total Crustacea**		**84**	**–**	**8**	**–**
**Mollusca – Gastropoda**					
**Patellidae**					
	*Patella candei*	38	0,3	35	2,5
	*Patella ordinaria*	3052	21	258	18,4
	*Patella piperata*	112	1	49	3,5
	*Patella ulyssiponensis*	2440	16,8	239	17,1
	*Patella* sp.	317	2,2	82	5,8
**Trochidae**					
	*Phorcus sauciatus*	1759	12,1	467	33,3
	*Phorcus* sp.	27	0,2	14	1
	*Gibbula* sp.	1	0	1	0,1
**Muricidae**					
	*Stramonita haemastoma*	1488	10,2	156	11,1
**Pisaniidae**					
	*Gemophos viverratus*	3	0	3	0,2
**Littorinidae**					
	*Tectarius striatus*	55	0,4	54	3,8
**Columbellidae**					
	*Columbella adansoni*	40	0,3	30	2,1
	*Mitrella moleculina*	1	0	1	0,1
**Conidae**					
	*Conus* sp.	3	0	2	0,1
**Cypraeidae**					
	*Cypraeidae* sp.	1	0	1	0,1
**Nassariinae**					
	*Tritia conspersa*	1	0	1	0,1
**Haliotidae**					
	*Haliotis tuberculata coccinea*	81	0,5	8	0,6
**Vermetidae**					
	*Thylacodes arenarius*	1	0	1	0,1
**Mollusca – Bivalvia**					
**Spondylidae**					
	*Spondylus senegalensis*	35	0,2	1	0,1
**Mollusca – Scaphopoda**					
**Dentaliidae**					
	*Antalis vulgaris*	5	0	5	0,5
**Total identified Mollusca**		**9460**	**100**	**1405**	**100**
**Unidentified remains**					
	Unidentified fishbones	631	–	–	–
	Unidentified molluscs	5048	–	–	–
**Total identified**		**13936**	**–**	**–**	**–**
**Total**		**19615**	**–**	**1498**	**–**

Twenty-seven bony-fish species in 12 families were identified (Table 2; Fig 4). Garfish (*Belone belone*) is dominant, comprising nearly 70% of the identified bones and 35% of the MNI, although fragmentation likely masks its true prevalence. Other common taxa include parrotfish (*Sparisoma cretense*), sardines (Clupeidae), *salema* (*Sarpa salpa*), and bogue (*Boops boops*). Less frequent finds include a tooth and vertebra of sandbar shark (*Carcharhinus plumbeus*). Numerous unidentified fish scales, particularly from SU 3, provide further evidence for on-site fish processing (Fig 4c). The remaining unidentified fish remains (n = 631) primarily consist of fragmented vertebrae and spines, while identified remains include bones from neurocranium, viscerocranium and axial elements such as parrotfish pharyngeals (Fig 4b).

### 3.5. Terrestrial fauna

A total of 1,057 terrestrial-mammal remains, 31 reptile bones, 2 avian shaft fragments, 12 indeterminate small-vertebrate elements, and 131 undiagnostic splinters were recovered. Rodent or carnivore bioturbation is minimal, although some insect traces were observed (54). The assemblage comprises two domestic taxa: goat (*Capra* *hircus*) and pig (*Sus scrofa domesticus*); and five wild taxa: house mouse (*Mus musculus*), rat (*Rattus* sp.), giant lizard (*Gallotia stehlini*), skink (*Chalcides* sp.), and gecko (*Tarentola* sp.) (Table 4).

**Fig 5:**
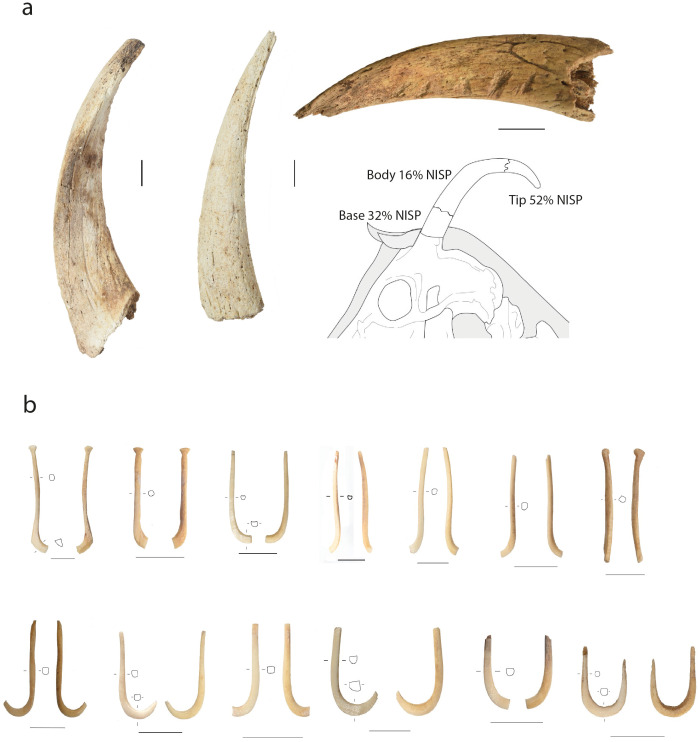
Worked terrestrial faunal remains from phase 5 in Zone 1, Playa Chica. **a)** goat horns (left), goat horn with chopping marks on one side (upper right), and anatomical distribution of goat horns from Phase 5 based on NISP; **b)** finished and fractured fishhooks made using pig tusks. The scale bars indicate 1 cm.

Goats are the most abundant mammal species, comprising approximately 79% of the NISP and a minimum of 40 adult individuals (Table 3). These individuals were primarily identified by the right horn core apices (*processus cornualis*, henceforth referred to as “horns”) (Table 4, Figs 3c and 5a). Additional caprine elements include 4 tooth fragments, 3 long bone splinters, and 1 vertebra lacking diagnostic traits (S1 Table).

**Table 3:** List of terrestrial fauna identified from Phase 5 in Zone 1, Playa Chica. MSM: medium-sized mammal; SVERT: small vertebrate; UNI: unidentified splinters.

Species	Common name	NISP	% NISP	MNE	% MNE	MNI	% MNI
*Capra hircus*	Goat	704	79	69	77	40	87
Caprines		8	1	4	4	1	2
*Sus s. domesticus*	Pig	127	14	17	19	5	11
*Mus musculus*	Mouse	4	<1	─	─	─	─
*Mus* sp.		36	4	─	─	─	─
*Rattus* sp.	Rat	1	<1	─	─	─	─
*Gallotia stehlini*	Gran Canaria giant lizard	6	1	─	─	─	─
*Gallotia* sp.		5	1	─	─	─	─
*Chalcides* sp.	Skink	2	<1	─	─	─	─
*Tarentola* sp.	Gecko	3	<1	─	─	─	─
**Total**		896		90		46	
**MSM**		177	─	─	─	─	─
**SVERT**		12	─	─	─	─	─
**BIRDS**		2	─	─	─	─	─
**REPTIL**		15	─	─	─	─	─
**UNI**		131	─	─	─	─	─
**Total**		337					

**Table 4:** Anatomical distribution of horn cores of *Capra hircus* from Phase 5 in Zone 1, Playa Chica.

Horn cores	NISP		MNE	MNI
**L**		**R**
Complete	4	8	12	8
Tip	25	32	57	32
Body	3	11		
Base	8	10		
Splinters (unidentified side)	603			
**Total**	**704**		**69**	**40**

Horn finds consist of 12 complete horns, 57 tips, 14 shaft fragments, 18 bases, and 603 splinters (Table 4, Fig 5a). The dominance of tips, coupled with the fact that 90% of the splinters originate from horn shaft knapping (NISP = 603/704), suggests deliberate modification (Fig 5a), likely to produce fish-descaling tools. Each specimen presents a bevelled distal edge employed to scrape scales; resharpening by secondary flake removal is visible on several of the implements. Both unretouched and freshly retouched examples are present in the deposit (Fig 5c). This hypothesis has been proposed for the similar horn finds recorded at the coastal site of Lomo Los Melones, also on Gran Canaria Island [31]. The lack of other animal body elements implies the intentional introduction of detached horns for fish processing and do not reflect activities of goat meat consumption at the site.

The pig is the second most prevalent taxon. Similarly to goat remains, it lacks anatomical evidence of meat processing (Supplementary S1 Table) since 86% of the 127 pig remains are fragments of lower canine enamel (NISP = 109/127) (Table 5). An intact lower canine and eight large fragments in SU 3 confirm that *Sus scrofa domesticus* is the taxon identified; no other native or domestic mammals on the Canary Islands possess canines that match the remains recorded at Playa Chica. At least five male tusks, identified by crown morphology [41,42] and two mandibular symphyses representing five adult individuals exhibit extensive working traces associated with fishhook production. In total, twenty-seven diagnostic fragments of pig-tooth fishhooks were recovered, along with 18 technologically modified pieces and numerous enamel chips (Figs 3d and 5b).

**Table 5:** Anatomical distribution data of *Sus s. domesticus* from Phase 5 in Zone 1, Playa Chica (L = left; R = right).

BONE		NISP			MNE	MNI
**L**			**R**	**Fragments with indeterminate side**
**Skull**	***Cranium***	1	1	2	1	1
	***Mandible***					
	Ramus	2	1			
	Body	3	3			
	Symphysis	2			2	2
	Splinters	–	–	2		
	***Isolated lower teeth***					
	Incisor	2	1	5	3	
	Canine	3	6	72	7	5
	Premolar	3	–	1	3	
	Splinters	–	–	16		
	**Subtotal**	126				
**Postcranial**	***Metatarsus I***			1	1	
	**Total**	**127**			**17**	**5**

**Fig 6:**
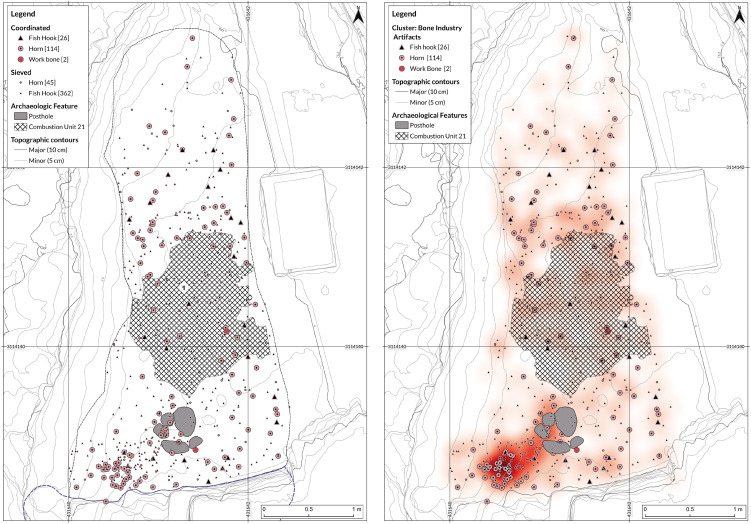
Spatial distribution of terrestrial faunal remains associated with the manufacture of enamel fragments for fishhooks and horns in phase 5 at Playa Chica. Specimens recorded in situ with three-dimensional coordinates are shown as large symbols, whereas additional remains recovered through sediment processing and subsequently spatially represented within their provenience polygons are shown as small symbols. Major archaeological features are also indicated. Left: coordinated and sieved enamel and horns artifacts or fragments. Right: the heat map depicts DBSCAN-derived clustering (ε = 0.15; minPts = 5) based on the full dataset (n = 545).

At this site, a large part of the *chaîne opératoire* of hook manufacture has been documented from tusk extraction through to the discard of worn hooks (Fig 5b). The finished hooks exhibit fine quality of manufacture, variable curvature, and a shank bearing a protruding knob produced by notching the labial edge. Fishhook debris concentrates in the southern sector of Zone 1, adjacent to the posthole and to the area rich in worked goat horns (Fig 6).

The DBSCAN analysis identified 18 statistically coherent clusters of goat and pig remains, with a moderate proportion of points classified as noise (16.9%; 92/545) (Fig 6). Cluster density is not homogeneous across the excavated surface. Most clusters are spatially anchored around the central combustion area (CS 21), consistent with repeated deposition and discard in the immediate vicinity of hearth-related activities. In contrast, the strongest material concentrations occur in the southern sector of Zone 1, where an exceptionally dense accumulation of fragmented and complete goat horns is tightly co-located with a posthole feature. The spatial coupling of a high-density horn concentration and a fixed architectural element supports a non-random, spatially structured patterning of deposition, suggesting a discrete activity locus within Phase 5 rather than diffuse discard.

The assemblage of wild terrestrial animals is limited to micromammals and reptiles. The sealed context and absence of burrows indicate that these animals entered the shelter contemporaneously with human activity [55]. House mouse is the most frequent wild taxon, represented by a broad array of skeletal parts, whereas a single rat axis vertebra was also recovered (Supplementary S1 Table). Rodent gnawing and other scavenging traces are negligible. Reptile remains are scarce and dominated by cranial elements; the endemic giant lizard is best represented, with skink and gecko present in smaller numbers.

### 3.6. Seeds and fruit remains

**Fig 7:**
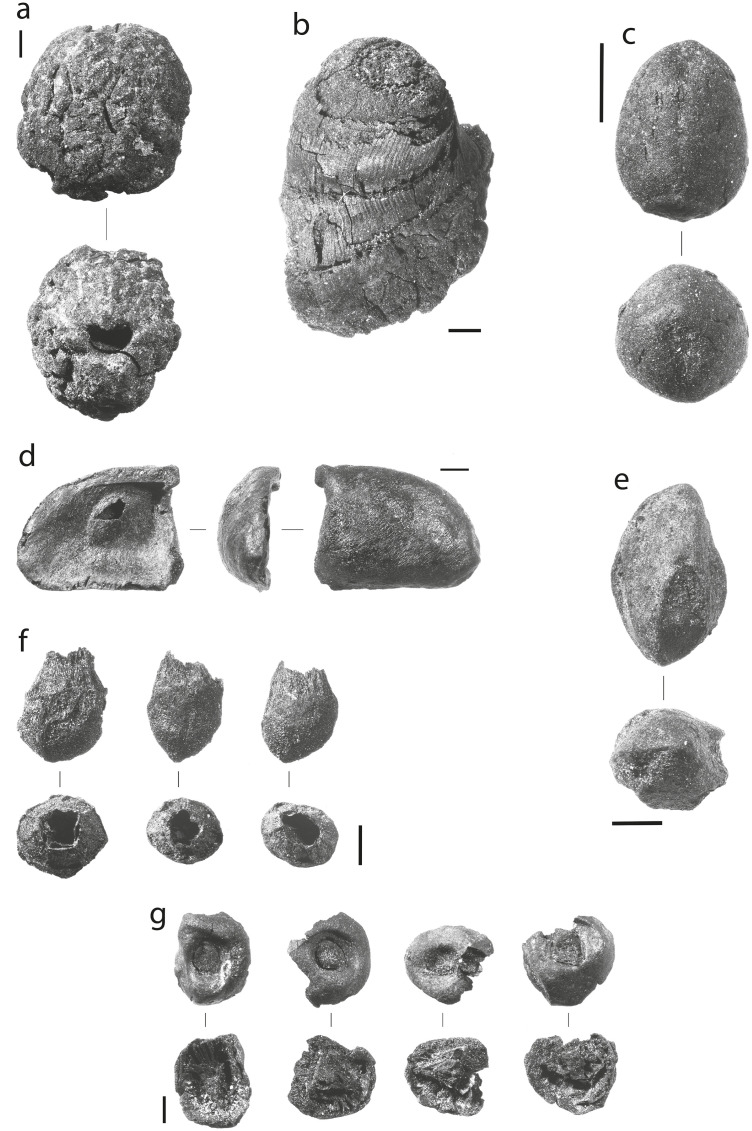
Charred seeds and other plant macro-remains from Phase 5 in Zone 1, Playa Chica. **a)** *Cneorum pulverulentum,* seed; **b)** cf. *Cyperus* sp., tuber fragment; **c)** *Euphorbia* sp., seed; **d)** *Euphorbia* sp. seed capsule; **e)** *Juniperus canariensis,* seed; **f)** *Pinus canariensis*, spur; **g)** *Phoenix canariensis*, perianth. The scale bars indicate 1 mm. The scale bars indicate 1 mm.

From 115 sediment samples analysed, 1,124 seeds remains were recovered, of which 1,051 were identified. The remains represent 27 taxa, including crops, native plants of the Canary Islands, and weeds (Table 6). Figure 7 shows images of the most abundant wild taxa.

**Table 6:** Charred seeds and other plant macro-remains from Phase 5 in Zone 1, Playa Chica.

Year	2020	2021	2022	Total	Percentage
**Number of samples**	54	43	18	115	
**Volume of sediment**	158	214	85	457	
**Crops**					
*Hordeum vulgare*, seed	5	3	2	10	0,89%
*Hordeum vulgare*, rachis segment	10		1	11	0,98%
*Triticum* cf. *durum*, seed			1	1	0,09%
Cerealia, seed	3	3	1	7	0,62%
*Ficus carica*, seed	284	197	14	495	44,04%
**Canary Islands native plants**					
*Cneorum pulverulentum,* seed	4	5		9	0,80%
cf. *Cyperus* sp., tuber fragment	17	17	2	36	3,20%
*Euphorbia* sp. seed capsule	24	12	2	38	3,38%
*Euphorbia* sp., seed	2		1	3	0,27%
*Juniperus canariensis,* seed	2	2	1	5	0,44%
*Juniperus canariensis*, leaf fragment	5			5	0,44%
Lauraceae, seed	1			1	0,09%
*Phoenix canariensis*, perianth	4			4	0,36%
*Phoenix canariensis*, seed			1	1	0,09%
*Pinus canariensis*, spur	33	25	2	60	5,34%
*Pinus canariensis*, seed scale	54	129	16	199	17,70%
*Pinus canariensis*, bark fragment	49			49	4,36%
*Pinus canariensis*, seed	1	3		4	0,36%
*Visnea mocanera*, seed		1		1	0,09%
**Weeds**					
Asteraceae, *capitulum*	1	1	1	3	0,27%
*Avena* sp., seed		1		1	0,09%
*Calendula* sp., seed	3			3	0,27%
*Chenopodium* sp., seed	4	5		9	0,80%
*Emex spinosa,* seed	4	4		8	0,71%
*Fumaria* sp., seed	43	23		66	5,87%
*Galium* sp., seed	1	3		4	0,36%
Lamiaceae, seed	1			1	0,09%
*Malva* sp., seed	4	2	1	7	0,62%
*Patellifolia* sp., seed		3	2	5	0,44%
*Phalaris* sp., seed		1		1	0,09%
*Plantago* sp., seed	1			1	0,09%
*Rumex* sp., seed	1			1	0,09%
*Silene gallica,* seed	1	1		2	0,18%
Indeterminate fragment	18	55		73	6,49%
**Total**	**580**	**496**	**48**	**1124**	

Crops are the most prevalent plant remains, with barley (*Hordeum vulgare*), wheat (*Triticum* cf. *durum*), and fig (*Ficus carica*) being documented. Figure is the most abundant crop, represented by 495 seeds. Barley is less common, with 10 seeds and 11 rachis segments recorded. Wheat is represented by a single seed, likely from durum wheat.

The Canary Islands’ native plants are also well-represented, with eight taxa identified. Pine (*Pinus canariensis*) is the most abundant, with 312 items, including seeds, seed scales, bark fragments, and spurs (Fig 7). Spurge (*Euphorbia* sp.) is present with 41 items, comprising seeds and seed capsules. Notably, tuber fragments likely belonging to *Cyperus* sp. are abundant, marking the first archaeological record of *Cyperus* rhizomes on the Canary Islands (Fig 7). Other native plants identified in Zone 1 are less common and include ‘leñabuena’ (*Cneorum pulverulentum*), juniper (*Juniperus canariensis*), laurel (Lauraceae), Canarian date palm (*Phoenix canariensis*), and ‘mocán’ (*Visnea mocanera*).

Weeds are less abundant compared to crops and native plants, with a total of 112 seeds recorded. Fourteen taxa have been identified, with most weed seeds belonging to *Fumaria* sp., accounting for 66 seeds. The remaining weed taxa are represented by fewer than ten seeds each.

### 3.7. Wood charcoal evidence

**Fig 8:**
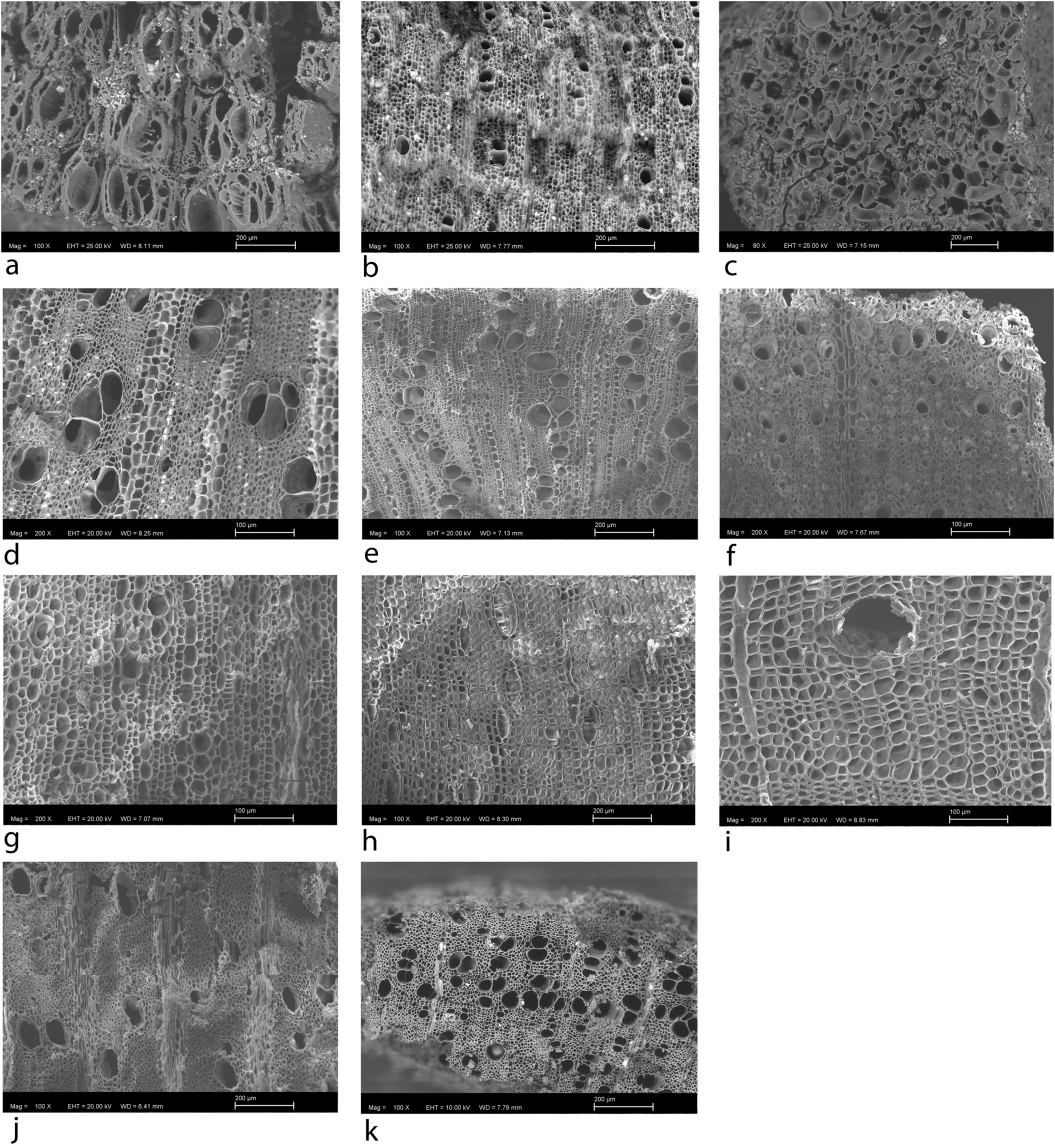
SEM photographs of wood charcoal remains. **a)** Chenopodiaceae, TS, x100; **b)** *Ficus carica*, TS, x100; **c)** *Pinus canariensis* seed scale, x80; **d)** Asteraceae, TS, x200; **e)** *Periploca laevigata*, TS, x100; **f)** *Erica arborea*, TS, x200; **g)** *Euphorbia* sp., TS, x200; **h)** *Juniperus canariensis*, TS, x100; **i)** *Pinus canariensis*, TS, x200; **j)** *Tamarix canariensis*, TS, x100; **k)** *Withania aristata*, TS, x100.

**Fig 9:**
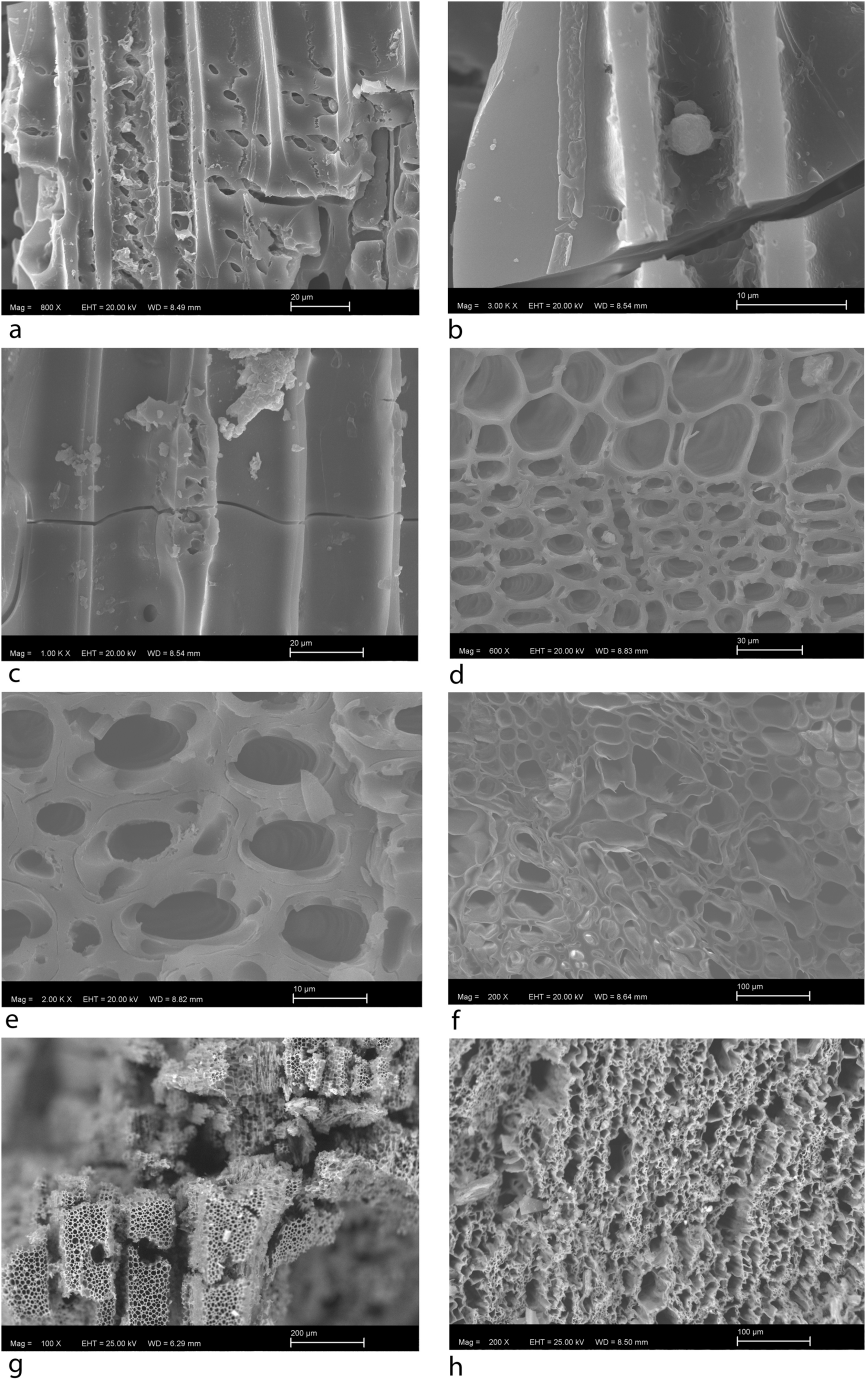
SEM photographs of wood charcoal remains with taphonomic affections. **a)** *Juniperus canariensis*, RS, x800: cavities in cell walls caused by hyphae; **b)** *Juniperus turbinata* ssp*. canariensis*, LRS, x3000: detail of fungal hyphae; **c)** *Juniperus canariensis*, LTS, x1000: fungal attack on ray cells; **d)** *Pinus canariensis*, TS, x600: preferential fungal attack on late wood; **e)** *Pinus canariensis*, TS, x2000: detail of cavities in cell walls (late wood); **f)** *Pinus canariensis*, TS, x600: advanced cellular deformation; **g)** *Tamarix canariensis*, TS, x100: fragmented tissue and galleries of wood-boring insects; **h)** *Euphorbia* sp., TS, x200: advanced cellular deformation.

Wood charcoal analysis in Zone 1 has so far focused on two combustion structures (CS 6 and CS 10) (Table 7). The number of charcoals remains recovered in the fireplaces is low, although the taxonomic diversity is high for concentrated anthracological assemblages (combustion structures) [56,57]. The woody taxa most commonly used as fuel in fireplaces are *Euphorbia* sp. (possibly *E. balsamifera* or *E. regis-jubae*), *Ficus carica*, and *Tamarix canariensis* (Figs 8 and 9). These results contrast sharply with the preferential use of Canary Island pine wood as fuel documented in most aboriginal sites [58]. Other taxa represented come from coastal plant formations and the thermophilic forest (Asteraceae, Chenopodiaceae, Fabaceae, *Lycium intricatum*, *Juniperus canariensis*, *Periploca laevigata*, *Pistacia atlantica, Pistacia lentiscus, Withania aristata*), the laurel forest (Lauraceae, *Erica arborea, Salix canariensis, Visnea mocanera*) and the pine forest (*Pinus canariensis*) (Figs 8 and 9). However, coastal scrubland is more prevalent than the other plant formation types mentioned due to its proximity to the site.

**Table 7:** Wood charcoal remains from Phase 5 in Zone 1, Playa Chica.

	CS 6		CS 10	
**Stratigraphic Unit**	18		2	
**Taxa**	n	%	n	%
Angiosperm	2	2,78	10	8,77
Asteraceae			8	7,02
Chenopodiaceae	2	2,78	1	0,88
Conifer			1	0,88
*Euphorbia* sp.	26	36,11	31	27,19
Fabaceae	1	1,39		
*Ficus carica*	11	15,28	21	18,42
*Juniperus canariensis*	1	1,39		
Lauraceae			1	0,88
*Lycium intricatum*	1	1,39	2	1,75
*Pinus canariensis*	2	2,78	3	2,63
*Pinus canariensis* (seed scale fragments)	1	1,39	8	7,02
*Pistacia atlantica*	5	6,94	1	0,88
*Pistacia lentiscus*			2	1,75
*Pistacia* sp.			2	1,75
*Plocama pendula*			2	1,75
*Salix canariensis*			1	0,88
*Tamarix canariensis*	16	22,22	1	0,88
*Visnea mocanera*			1	0,88
*Withania aristata*	1	1,39		
Parenchyma tissue	3	4,17	18	15,79
*Total*	72		114	

### 3.8. Artifacts

**Fig 10:**
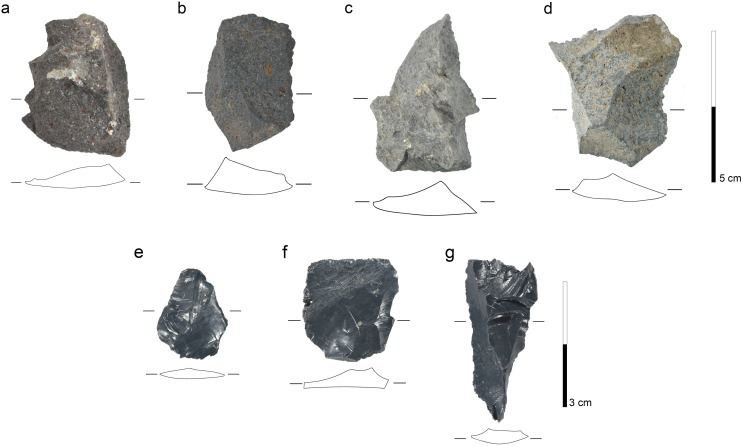
Knapped lithics from Zone 1 at Playa Chica. **a)** pla21_63, basalt flake with left lateral denticulate retouch; **b)** pla20_119, unidirectional basalt flake; **c)** pla21_103, unidirectional trachyte flake; **d)** pla21_120, centripetal basalt flake; **e)** pla20_40-2, bipolar trachytic obsidian flake; **f)** pla21_305, *esquillée* trachytic obsidian flake; **g)** pla21_518, bipolar trachytic obsidian flake.

Phase 5 yielded 10,166 lithic artifacts, manufactured mainly from coarse volcanic rocks (basalt, phonolite), with smaller quantities of obsidian and other siliceous rocks (Table 8). The high proportion of waste flakes, together with stone hammers and anvils, indicates on-site knapping (Fig 10a–10d). Most large flakes are cortical or semi-cortical removals produced during early cobble and block reduction rather than from complete *débitage* sequences, consistent with reduction strategies documented at other Gran Canaria coastal sites [28,59]. Pronounced bulbs of percussion point to a direct hard-hammer technique.

**Table 8:** Lithic artifacts from Phase 5 in Zone 1, Playa Chica.

Supports		Coarse-grained volcanic rock	Obsidian	Other siliceous rock
**Chipped**	**Flakes**	673	20	47
	**Retouched flakes**	61	10	3
	**Other knapped tools**	20	–	–
	**Nucleus**	3	2	4
	**Debris**	8,655	470	127
**No chipped**	**Abrasive tools**	30	–	–
	**Percussion tools**	31	–	–
**Total amount**		9,473	502	181
**Total weight (gr)**		40,883	40,12	90,56

Coarse volcanic rock cobbles were collected locally from the Playa Chica shore and adjacent ravines, whereas obsidian derives from more distant sources. Two volcanic-glass varieties are present: (1) trachytic obsidian procured from primary outcrops on the island’s western flank (Fig 10e–10g); and (2) phonolite obsidian, represented by clasts likely obtained from detrital deposits in the south and southwest of Gran Canaria. Other siliceous rocks occur only as sparse cobbles or thin seams within lava flows. Owing to their small natural dimensions, obsidian and these siliceous cores were reduced by bipolar percussion: the nucleus was set on a stone anvil and struck from above, producing opposed flake negatives.

Non-flaked stone tools comprise hammers, anvils, and abrasive slabs that may have been used in fishhook manufacture. A subset of knapped implements was selected for use-wear analysis; however, most surfaces display post-depositional damage from trampling, heat alteration, and sediment abrasion, limiting functional interpretation [60].

**Fig 11:**
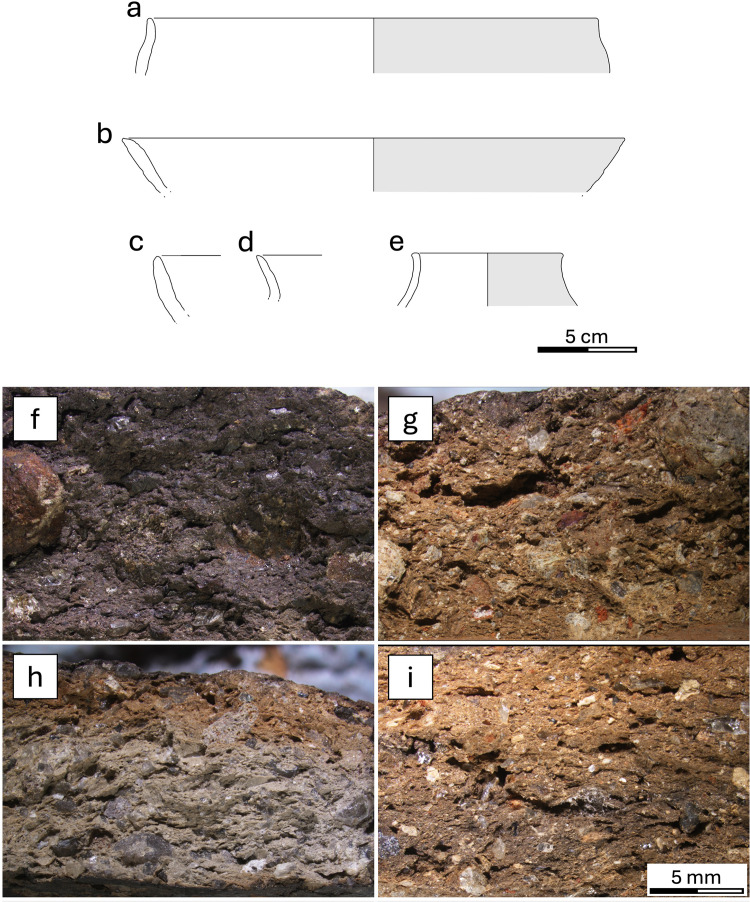
Pottery from Zone 1 at Playa Chica. **a–e)** Pottery shapes identified in Playa Chica; **f–h)** Main macroscopic fabrics; **i)** An individual of the second fabric exhibiting possible secondary calcite crystallization.

The assemblage comprises fifteen fragments of ceramic sherds (Table 9). Most exhibit severe surface erosion, likely the consequence of post-depositional processes such as sediment mobility in sandy environments and fluctuating moisture levels. Given the high degree of fragmentation and the scarcity of diagnostic pieces (Fig 11a–11e), no attempt is made to estimate the original vessel count. Prior petrographic studies have identified considerable homogeneity in local ceramic pastes on Gran Canaria, further restricting such reconstructions.

**Table 9:** Ceramic individuals from Phase 5 in Zone 1. The firing atmosphere describes the coloration of the sherds’ section: R: dark-reducing, O: light-oxidizing (after Day and Kilikoglou [61]).

Specimen	SU	Thickness (cm)	Decoration	Firing atmosphere	Surface erosion	Typology
PLA20/20	1	0.8		R	x	I
PLA20/6.1	1	0.9		R		I
PLA20/6.2	1	0.9		R	x	Ia
PLA20/62	1	0.8	painting/slip	O	x	II
PLA20/1713	2	1.0		ROR	x	I
PLA20/126–2	2	0.9		O	x	II
PLA20/1708	2	1.3		O-R	x	II
PLA20/171–1	2	1.3		ORO	x	II
PLA21/58	2	0.4	painting/slip	O	x	II
PLA21/148–5.1	2	0.9		ORO	x	II
PLA21/148–5.2	2	0.8		ORO	x	II
PLA21/356–3	5	1.5		ORO	x	II
PLA21/378–5	5	0.5		ORO		II
PLA21/509–5	5	0.9		ORO		II
PLA21/295–4	2	0.4		O	x	II

Technologically, the material is consistent with Indigenous‑period ceramics from the Canary Islands. All sherds are handmade in medium‑ to coarse‑grained pastes and exhibit irregular firing, as indicated by colour contrasts between surfaces and cores (Fig 11f–11i). Wall thickness ranges from ~0.5 cm to 1 cm. Only a single rim fragment provides morphological information: it belongs to a tray that bears exterior traces of direct heat exposure and fits typological subgroup 1a of the Gran Canaria series [45] (Fig 11a, 11b). Open‑form cooking trays of this type are widespread and most associated with grain roasting, though experimental and contextual evidence suggests they could also have been used to process other foods, including in ritual settings (e.g., El Tejar). Four additional body sherds display comparable thermal alteration, implying that they too derive from cooking vessels.

The remaining pieces probably fall within the heterogeneous Group II of del Pino et al. [45], whose functions range from consumption to storage (Fig 11e). Only two fragments show surface decoration: both are treated with *almagre* (red ochre) pigment and burnishing. On item PLA20/62, the incomplete pigment field suggests a geometric motif, one of the island’s most common decorative schemes. Although the scarcity of decorated sherds may partly reflect surface erosion, it is noteworthy that similar late contexts in the Gáldar area typically yield higher proportions of ornamented ceramics. In this respect, the Sardina assemblage resembles those from specialized coastal sites such as Dunas de Maspalomas, where ceramic inventories are limited and dominated by cooking trays [28].

## 4. Discussion

Archaeological investigations at Playa Chica have significantly advanced our understanding of marine-resource exploitation on Gran Canaria during the Indigenous (pre-European) period. Located on the island’s northern coast, the site preserves a five-phase cultural sequence spanning the 6^th^ to 13^th^ centuries CE (Table 1). Although archaeological works at Playa Chica are part of an ongoing project, current archaeological evidence suggests that in the earlier phases (1–4), inhabitants practiced a mixed subsistence economy that combined fishing, small-scale agriculture, and animal husbandry. Between the 11^th^ and 13^th^ centuries CE (Table 1), however, evidence suggests that the site became increasingly specialized in the collecting and processing of marine products, representing a critical shift toward a more intensified coastal economy.

A substantial stone wall situated along the southern boundary of Zone 1 likely served as a windbreak against prevailing northerly and north-easterly winds. An associated post-hole indicates the presence of a superstructure, which could be either a shelter providing workspace protection or a facility dedicated to fish processing. Faunal assemblages within Zone 1 exhibit dense concentrations of fish and shellfish, while specialized implements such as pig-tusk fishhooks and goat-horn scalers are also abundant. Numerous flat and shallow pit hearths with repeated ignition episodes are clustered with these artifacts, supporting the interpretation of Zone 1 as a dedicated area for the capture and processing of marine resources.

### 4.1. Exploitation of marine resources

The ichthyofaunal assemblage from Playa Chica reflects reliance on small pelagic and demersal species that were probably collected at the waters of Sardina Bay. Most of the taxa can be found nowadays in rocky and sandy coastal areas of Gran Canaria. Large pelagic taxa from open waters such as tuna are not recorded, with one shark tooth being the only example of this type of fish. This pattern, dominated by inshore taxa with minimal evidence of offshore exploitation, aligns with models of nearshore-focused maritime adaptations documented in other island societies during formative stages of coastal economy development [2,4]. While these data support earlier suggestions that fishing was primarily shore-based [62], the absence of pelagic taxa does not necessarily preclude all watercraft use but rather indicates that marine exploitation strategies remained oriented toward the highly productive intertidal and nearshore zones characteristic of early island maritime economies [1].

The taxa recorded at Playa Chica has been identified in previous studies on other coastal sites of Gran Canaria [28,62]. However, at Playa Chica the amount of remains from garfish (*Belone belone*) is much higher than in other sites of the island. This difference may be since in Playa Chica all the sediments were sampled and processed with 1 mm sieves, allowing the recording of small fish bones, such as the ones from garfish (*Belone belone*). Nevertheless, we cannot rule out that garfish was more abundant in this part of Gran Canaria coast than in other parts of the island.

In relation to fish capture techniques, although no direct archaeological evidence of nets was recovered, an expected gap given the perishable nature of organic fibre, ethnohistoric textual records [62] and the taxonomic composition of the ichthyofaunal assemblage itself, provides strong indirect evidence for their use. The abundant representation of small-bodied, schooling species such as sardines (*Sardina pilchardus*) and bogues (*Boops boops*) points to the deployment of nets, as these taxa cannot be efficiently captured by hook and line [31,62]. Fine-mesh nets would have been particularly effective for these species, as suggested by historical narratives describing the capture of sardines and mullets with nets.

Ethnographic data from Gran Canaria and the wider Macaronesian region indicate that seine nets (large devices with floats and weighted footropes) were traditionally deployed in sheltered bays to encircle schools of fish. The calm waters and constraining coastal topography of Sardina Bay would have facilitated such hauls. Cast nets, smaller hand-thrown devices used from shore, would likewise have been effective for intercepting fish near the rocky coastline [63,64]. Zooarchaeological evidence from other pre-European sites in the Canary Islands suggests similarly extensive use of nets [31,62].

Line fishing was also practiced, as indicated by the numerous fishhooks recovered at Playa Chica. Their size suggests they targeted small- to medium-sized fish caught from the shoreline, including garfish (*Belone belone*), parrotfish (*Sparisoma cretense*), salema (*Sarpa salpa*), and bogue (*Boops boops*). In this case, hook size appears to be constrained not by the intended catch but by the manufacturing raw material (pig tusk). Comparable artifacts have been recorded at other sites in Gran Canaria such as Aguadulce, El Llanillo, Llano de Las Brujas, Cueva Pintada, Cendro, Lomo Los Melones, and La Restinga (Fig 1).

The hooks from Playa Chica are J-shaped jabbing hooks that require a sharp pull to secure the catch. Analogous objects, though made from horn keratin sheaths, are known from the western islands, albeit without archaeological context [65]. However, their morphology appears better suited to functioning as hanging hooks rather than as line hooks [62]. Moreover, a double-pointed implement from La Palma (Cueva Chica, Puntallana) indicates that comparable fishing gear may also have been used on other islands [66].

Playa Chica has yielded the largest corpus of Indigenous-period hooks on Gran Canaria and is the only site with clear evidence of their manufacture. These hooks would have been well suited for catching garfish, a taxon also documented at hook-bearing sites such as Cendro [62]. Additionally, it is possible that garfish remains may have been underrepresented in earlier excavations due to the recovery and sorting procedures employed. The consistent presence of hooks across the site, underscores line fishing as a systematic, not incidental, activity.

The collection of shellfish within Sardina Bay is also well-documented. The most prevalent taxa, primarily gastropods, inhabit rocky and sandy substrates along the intertidal zone. The dominant species, *burgao* (*Phorcus sauciatus*), thrives in the upper intertidal zone, where basaltic rock pools are submerged during periods of heavy surf and strong tides. This pattern contrasts with the malacological assemblages documented in shell middens from northwestern Tenerife, where limpets (*P. ordinaria* and *P. ulyssiponensis*) overwhelmingly dominate the record and top shells represent less than 2% of the MNI [40]. These inter-island differences may reflect local variations in intertidal substrate, wave exposure, or culturally mediated preferences in gathering strategies. While limpets and periwinkles were most likely gathered primarily for consumption at Playa Chica, shells such as cowries and columbellids may have been collected for ornamental or symbolic purposes, potentially with additional use as fishing lures. Heat-altered shells, characterized by discoloration and adhering wood charcoal, provide evidence that fire was integral to cooking and possibly preserving shellfish.

### 4.2. Specialized fishing and processing tools

Goats and pigs, two of the four domesticates exploited during the Indigenous period, were mainly used at Playa Chica as sources of raw materials rather than food. Goat horns and pig tusks were systematically transformed into fish scalers and hooks, respectively, with manufacture clearly taking place on site.

The production of pig-tusk hooks required considerable skill: tusks had to be shaped and sharpened while preserving their structural integrity to withstand the tension of the line. Zone 1 has yielded production debris, partially worked blanks, and fractured hooks, pointing to a specialized craft tradition and the economic centrality of fishing. The absence of complete hooks suggests that finished examples either broke during the final stages of manufacture or fractured in use, returning to the site attached to catches or lines.

The recovery of isolated male pig tusks and mandibular portions, without accompanying butchery refuse, suggests that primary processing occurred elsewhere, with only suitable portions transported to Playa Chica. Insect traces on some mandibular remains support this interpretation [67]. This pattern suggests inter-site mobility and structured exchange networks, with male tusks circulating as valuable raw materials. Such technological specialization and material circulation are hallmarks of mature island economies, where marine resources provide not only subsistence but also surplus and social value that underpin broader exchange systems [3,5].

Goat horns are particularly abundant, representing approximately 90% of the goat remains from Zone 1, and occur in every stage of modification. Their contexts and morphology indicate their use as fish scalers or scrapers, a conclusion reinforced by the thousands of fish scales recovered. Comparable tools, sometimes still bearing scales, have been documented at Lomo Los Melones and El Burrero [31].

Micromammal and reptile bones reflect opportunistic shelter use by wild fauna attracted to fish-processing residues. The rarity of rodent gnawing on terrestrial bones further supports their primary role as a tool raw material rather than food waste.

Additional material culture reinforces the specialized character of Playa Chica. Ceramics are scarce and typologically uniform, paralleling coastal exploitation sites such as Dunas de Maspalomas [28]. Identified vessels consist mainly of cooking pots and small containers for food consumption, in contrast to the diverse decorated assemblages typical of domestic contexts. Zone 1 yielded only open, unrestricted cooking pots. This form, widespread from the 7^th^ century CE at La Cerera (Arucas) and present at settlements such as Cueva Pintada, El Tejar, San Antón, and Dunas de Maspalomas [21,26,52], has often been compared to traditional cereal roasters linked to *gofio* (roasted barley flour) production [16]. However, their broad distribution and the absence of milling equipment at Sardina suggest alternative functions that warrant further investigation.

The lithic assemblage further supports the interpretation of Zone 1 as a specialized activity area. The abundance of coarse volcanic flakes and debitage, together with hammers and anvils, indicates on-site tool production oriented toward immediate use rather than the manufacture of curated implements. The predominance of cortical and semi-cortical removals, rather than complete reduction sequences, suggests expedient knapping strategies consistent with task-specific needs [59,60]. Abrasive slabs may have served in the manufacture of bone fishhooks, linking lithic and osseous tool production within a unified technological system. The presence of obsidian from the island’s western outcrops demonstrates that Playa Chica was integrated into broader networks of raw material circulation, even though most stone tools were produced from locally available cobbles. This pattern of local expedient production combined with selective use of higher-quality materials mirrors strategies documented at other coastal sites in the archipelago and underscores the site’s role within island-wide exchange systems [28,58].

### 4.3. Plant use

Charred seeds, fruit fragments, and wood charcoal from Zone 1 at Playa Chica provide key evidence for reconstructing past environments and patterns of botanical resource use.

Many of the plant species identified through the analyses of seeds in Zone 1 at Playa Chica are native to the island’s coastal environments and still grow nearby today such as spurge (*Euphorbia* sp.) and ‘leñabuena’ (*Cneorum pulverulentum*). However, some taxa, such as juniper (*Juniperus canariensis)*, are no longer present in the immediate vicinity but may have been available in the past. Other species, including Canarian pine (*Pinus canariensis*), ‘mocán’ (*Visnea mocanera*) and ‘laurel’ (Lauraceae), which need higher humidity and grow in the mountains, were likely transported to the site.

In terms of economic activities, the seed assemblage from Zone 1 at Playa Chica indicated the performance of plant food processing, and possible consumption. This activity is evidenced by the presence of crops plants such as barley, wheat, and fig, in addition to wild native edible plants such as ‘mocán’ (*Visnea mocanera*) and Canarian date palm (*Phoenix canariensis*). Those taxa are the most common plants recorded in settlement sites and cave granaries of Gran Canaria [68], suggesting they were a frequent food for the indigenous population. Nevertheless, they are less abundant in Zone 1 than in Zone 2, where a preliminary study has also identified broad bean (*Vicia faba*) and lentil (*Lens culinaris*) [16].

The charred seed assemblage from Zone 1 at Playa Chica is equally distinctive and diverges markedly from Zone 2 and those typically found at settlement sites. Crops plants such as barley and figs are the most frequent and abundant botanical records at sites from Gran Canaria; on the contrary, wild plants are rare [16,64]. However, in Zone 1 of Playa Chica the seed assemblage includes several wild taxa that have not been recorded previously in indigenous sites of the Canary Islands such as the seeds and capsules of spurge (*Euphorbia* sp.) and rhizomes of ‘junquillo’ (*Cyperus* sp.). Additionally, this is also the only site in which pine spurs have been identified, and the abundance of pine seed scales, a component of the pinecone, is also distinctive, since they are infrequent in sites from Gran Canaria. Notably, the high representation of pinecone elements (seed scales, spurs, and bark fragments) contrasts sharply with the near-absence of *Pinus canariensis* wood charcoal in the analysed combustion structures (NISP = 5), where coastal scrubland taxa dominate. This disproportion suggests that pine was not utilized as firewood at Playa Chica, unlike the pattern observed at most inland settlement sites on the island [58]. Instead, pinecones and bark were selectively collected and transported to the site. When burned, pinecones and needles produce copious, low-temperature smoke, a property that aligns with the other fuel taxa identified at the site (*Euphorbia* sp., *Ficus carica*), all of which share a tendency to generate smoke rather than sustained high-temperature flames. The deliberate, selective transport of pinecone elements to a coastal site thus reinforces the interpretation that fuel procurement at Playa Chica was functionally oriented toward smoke production for fish preservation.

Similarly to the results of the seed analysis, the taxonomic spectrum of the wood charcoal differs markedly from domestic Indigenous contexts, where montane woods such as Canarian pine (*Pinus canariensis*) dominate [58]. The high diversity identified in the analysed wood charcoal samples (5–13 different species) can be interpreted in two possible ways: (1) as evidence of distinct but overlapping combustion events, reflecting prolonged use of the hearth over time (with refiring leading to the accumulation of fuel remains from successive burning episodes), or (2) as the result of firewood collection practices focused on gathering dead and dry wood from the surrounding environment (Fig 7), with selection based primarily on branch diameter (small-calibre branches) or state (fallen, dead wood), rather than on strict taxonomic preference. Such gathering pattern would explain the low representation of certain woody taxa [58]. However, the assemblage also suggests a degree of deliberate selection: firewood collection at Playa Chica appears to have concentrated on locally available dead branches, sometimes colonized by fungi or insects, as in the case of juniper and Canarian pine, and particularly on taxa with spongy wood (e.g., *Euphorbia*, *Ficus carica*), which may have been specifically targeted for their ability to produce smoke. These practices are consistent with the generation of embers rather than open flames, possibly indicating smoking-related activities.

Tentatively, we have interpreted the presence of seeds and wood charcoal of those plants as result of their use in the processing of marine foods, specifically for heat and smoke treatment. Spurge (*Euphorbia* sp.) and fig (*Ficus carica*), the most abundant taxa in the seed and wood charcoal analyses, are plants with sponge-like wood that is filled with latex, and they are not particularly suited for generating high-intensity, high-temperature fires. Instead, they produce short-lasting, smoke-heavy flames. This is also the case for ‘junquillo’ (*Cyperus* sp*.*) rhizomes, that were traditionally used in Lanzarote Island to heat and smoke the food [69]. *Cyperus* rhizomes are edible after processing and it is also possible, they were consumed, although there is no ethnographic evidence of such activity in the Canary Islands. Pinecones and leaves, the former evidenced by the presence of spurs, also produce high amounts of smoke.

By slowly drying and lightly smoking fish, and possibly shellfish, people would have extended the shelf life of marine products. Smoking and toasting would have reduced the moisture content of the fish flesh, as well as the presence of pathogens, mitigating spoilage and enabling storage or trade with inland communities [70]. Such preservation techniques are well-documented in island contexts worldwide, where they enable not only food security but also participation in exchange networks that connect coastal and inland communities [3,4].

The low-intensity combustion features found at the site reinforce this interpretation. As described in the Results section, the hearths in Zone 1 are flat or shallow, irregular pits fires of thin lenses of charcoal and ash, deposited directly on the sandy substrate or on other hearths, without stone lining or prepared surfaces. The hearths in Zone 1 are flat fires or located in small, irregular pits on the sand without specific preparation. Unlike some domestic hearths in Gran Canaria, these fires do not appear to have functioned as centralizing elements of domestic space. Instead, their morphology and placement are consistent with expedient, task-oriented fires designed for low-temperature combustion and smoke generation rather than for sustained domestic heating or cooking.

The posthole recorded in square 131C within SU 3, spatially associated with the main combustion area (CS 21), may represent part of a light wooden framework such as a rack or drying structure erected above or beside the hearths. Ethnographic and archaeological parallels suggest several plausible configurations for such processing. In traditional West African fish smoking, the simplest and most widespread technique involves laying fish directly on wooden racks or branches positioned above a shallow ground-level fire, a method that requires no permanent infrastructure and leaves minimal archaeological traces beyond the fire itself [71]. Similarly, among Pacific Northwest coastal communities, fish were either suspended from horizontal poles within light smokehouses or skewered on wooden stakes arranged around open pit fires [72]. At Playa Chica, the most parsimonious reconstruction envisages fish (and possibly shellfish) laid on simple branch frameworks or suspended from poles above smouldering fires of *Euphorbia* sp*.* and *Ficus carica*, fuels that, as noted above, produce abundant smoke at low temperature. Direct placement on embers cannot be ruled out for small items such as limpets, which ethnographically are often toasted directly on hot coals or ashes to detach the flesh from the shell [73]. However, for larger fish such as garfish and parrotfish, a rack or suspension system would have been necessary to maintain controlled, prolonged exposure to smoke without charring the product

In the early 20^th^ century, some coastal communities on Gran Canaria still used low‑intensity fires to heat and smoke fish, which they subsequently exchanged for crops grown by upland communities (Susa Jiménez Jiménez, pers. comm.). Though now rare, these techniques persist locally and shed light on practices at Playa Chica. Such adaptations would have buffered seasonal shortfalls, allowed surplus stockpiling, and facilitated participation in broader exchange networks.

### 4.4. The Canarians and the sea

Playa Chica’s marine exploitation between the 11t^h^ and 13^th^ centuries CE parallels archaeological contexts documented at other Gran Canarian coastal sites, including La Puntilla and Lomo Caserones [48,74]. On the southern coast, the archaeological site of Dunas de Maspalomas provides evidence for seasonally focused fishing and shellfish gathering between the 8^th^ and 11^th^ centuries CE [28]. On the eastern coast, structure 7B at La Restinga, radiocarbon dated to the 12^th^–13^th^ centuries CE, also yielded abundant fish and shellfish remains consistent with a specialized processing area [75]. A comparable context is found a few kilometres to the south at Lomo Los Melones, where fish and shellfish remains, as well as worked goat horns, have been recorded inside a stone-built structure dated to the 14^th^–15^th^ centuries CE [29,31].

Despite these parallels, Playa Chica stands out for the density and diversity of its specialized fishing implements. Neither Dunas de Maspalomas nor La Restinga produced substantial concentrations of fishhooks or associated toolkits, whereas Playa Chica preserves an unparalleled assemblage of hooks together with goat horns directly linked to fish remains. Worked goat horns are also documented at Lomo Los Melones, but the overall abundance and variety of materials there is far lower. These disparities may partly reflect recovery and preservation biases as Playa Chica was excavated with systematic sediment sampling, as well as local differences in resource availability, settlement organization, or the economic roles of individual sites within island exchange networks.

The shift toward intensified marine exploitation at Playa Chica coincides with broader demographic and socioeconomic changes on Gran Canaria during the 10^th^ and 11^th^ centuries CE [22,38,76–78]. Archaeological evidence, such as the expansion of cave-granaries and the growth of coastal settlements, suggests a population increase accompanied by efforts to improve food security [22,79–81]. Settlement aggregation along the shoreline is likewise documented for this period [38,82]. In this context, surplus fish and shellfish would have served both as dietary supplements and as commodities exchanged with inland communities.

Bioarchaeological data reinforce this interpretation. Skeletal assemblages from coastal sites exhibit elevated frequencies of auricular exostoses (bony growths in the ear canal associated with repeated immersion in cold water) after the 10^th^ century CE, suggesting intensified fishing or diving activities [38]. Stable-isotope analyses of 12^th^–14^th^-century individuals likewise show higher δ¹⁵N values, consistent with increased marine protein intake [32]. Taken together, these lines of evidence underscore the central role of marine resources in the subsistence strategies of the Indigenous population of Gran Canaria.

A comparable model of specialized coastal production has been proposed for the shell middens of Isla Baja in northwestern Tenerife, where long-term, occasional shellfish processing spanning from the 2^nd^ to the 12^th^ centuries CE appears to have been oriented toward deferred consumption by inland populations [40,83]. These middens show stable mollusc size through time, consistent with sustainable exploitation, and their taxonomic assemblages differ from those of residential sites, where taxa are more diverse, a pattern that parallels the distinction between specialized and domestic contexts at Playa Chica.

## 5. Conclusions

Archaeological research at Playa Chica has documented a stratified sequence of five occupation phases spanning from the 6^th^ to the 13^th^ centuries CE. The four earliest phases (6^th^–10^th^ centuries CE) attest to domestic activities carried out inside a stone structure, while the most recent phase (Phase 5, 11^th^–13^th^ centuries CE) reveals a marked functional shift toward the intensive extraction and processing of marine resources. The multiproxy analysis of Phase 5 in Zone 1 offers a well-documented case of specialized coastal exploitation, a topic that remains poorly understood in other African coastal contexts, particularly in western North Africa. This multi-proxy approach provides a methodological framework applicable to other understudied coastal contexts in the Canary Islands and the broader African Atlantic.

Collectively, the concordance across all lines of evidence establishes that Zone 1 of Playa Chica functioned during Phase 5 as a dedicated area for the capture, processing, and likely preservation of marine resources. The density and diversity of marine fauna, the presence of a specialized bone industry, and the systematic use of smoke-producing fuels define an activity area that contrasts markedly with contemporaneous domestic and craft-production contexts documented elsewhere on Gran Canaria. These results suggest that Playa Chica supplied fish, shellfish, and preserved seafood to neighbouring households or inland communities, pointing to the integration of coastal exploitation within broader exchange networks during the 11^th^–13^th^ centuries CE. The resulting picture is of a focused, perhaps communal, activity area that generated surplus marine products, a pattern consistent with mature island economies where coastal resources provide not only subsistence but also commodities for exchange and social value.

Nevertheless, limited data from the earlier phases at Playa Chica and the scarcity of systematically excavated coastal sites across the archipelago render these conclusions preliminary. Recent work on shell middens and soil terraces in Tenerife has demonstrated that multi-proxy approaches to coastal archaeological deposits can successfully reconstruct indigenous subsistence practices, underscoring the need for comparable systematic investigations across the Canary Islands. These gaps echo broader challenges in African coastal archaeology, where subsistence records from coastal sites remain fragmentary. Future research should prioritize the excavation and fine-grained sampling of additional coastal contexts to establish whether comparable patterns of specialized marine exploitation existed elsewhere.

The evidence from Playa Chica illuminates the long-term processes by which Berber populations from Northwest Africa developed sophisticated coastal adaptations following their arrival in the Canary Islands. This trajectory mirrors patterns documented in other island contexts globally, where the structural constraints of limited terrestrial biomass, combined with the high productivity of coastal ecosystems, drove the gradual intensification of marine exploitation. The findings from Playa Chica provide compelling evidence that the Canarian Sea was not a marginal resource exploited solely out of necessity, but rather a central social space where specialized practices materialized. In this context, Playa Chica does not represent an anomaly but rather the archaeological manifestation of a broader pattern: the construction of the coastline as a productive interface where the Berber populations of Gran Canaria articulated their subsistence, their seasonal calendar, and, presumably, their collective identity.

As the only Atlantic archipelago colonized by Berber populations during the late Holocene, the Canary Islands provide critical comparative data for broader debates on the development of coastal economies in Africa, a region where, despite extensive coastlines, maritime adaptations remain remarkably understudied. This is particularly relevant in light of the limited archaeological and bioarchaeological evidence for coastal adaptations among western North African populations during the Late Holocene. Playa Chica demonstrates that detailed, multi-proxy investigations of island coastal sites can reveal the nuanced, long-term processes by which human societies transformed their relationship with the sea.

## Supporting information

S1 Table Zooarchaeological assemblage composition by taxon and skeletal element at Zone 1 in Playa Chica. The table reports the number of identified specimens (NISP) for each anatomical element across domestic, wild, and synanthropic taxa identified in the assemblage, including mammals and reptiles. Indeterminate fragments and taxonomically ambiguous categories (e.g., Capra/Ovis) are reported separately. Blank cells indicate absence of identified remains.
(XLSX)
